# *Akkermansia muciniphila* Ameliorates Alcoholic Liver Disease in Experimental Mice by Regulating Serum Metabolism and Improving Gut Dysbiosis

**DOI:** 10.3390/metabo13101057

**Published:** 2023-10-07

**Authors:** Cheng Fang, Jinyan Cheng, Wei Jia, Yan Xu

**Affiliations:** 1 Laboratory of Brewing Microbiology and Applied Enzymology, School of Biotechnology, Jiangnan University, Wuxi 214122, China; cfang@jiangnan.edu.cn (C.F.); 6210208006@stu.jiangnan.edu.cn (J.C.); 2 Key Laboratory of Industrial Biotechnology, Ministry of Education, School of Biotechnology, Jiangnan University, Wuxi 214122, China; 3 Center for Translational Medicine, Shanghai Jiao Tong University Affiliated Sixth People’s Hospital, 600 Yishan Road, Shanghai 200233, China; weijia1@hkbu.edu.hk; 4 School of Chinese Medicine, Hong Kong Baptist University, Kowloon Tong, Hong Kong, China

**Keywords:** *Akkermansia muciniphila*, gut microbiota, serum metabolome, ALD

## Abstract

Alcoholic liver disease (ALD) represents a significant global health concern, yet the available treatment options remain limited. Numerous studies have shown that gut microbiota is a critical target for the treatment of ALD. Additionally, there is increasing evidence that host metabolism also plays a crucial role in the development of ALD. *Akkermansia muciniphila* has been demonstrated to ameliorate experimental ALD through its modulatory effects on the intestinal vascular barrier, enhancement of mucus layer thickness, and promotion of intestinal tight junction proteins. Nevertheless, there is a dearth of studies investigating the impact of *A. muciniphila* on host metabolism and gut microbiota. Here, C57BL/6 mice were utilized to establish a modified NIAAA model in order to investigate the impact of the oral administration of *A. muciniphila* during the development of ALD. Furthermore, we employed targeted metabolomics to analyze the serum metabolomic profiles of the mice and 2bRAD-M sequencing to comprehensively examine the underlying mechanisms of the efficacy of *A. muciniphila* on ALD. Our results illustrated that the oral administration of *A. muciniphila* alleviated alcohol-induced liver injury in conjunction with encouraged serum levels of ornithine and diminished the elevation of oxalic acid levels induced by alcohol intake. In addition, *A. muciniphila* also inhibited the proliferation of harmful bacteria, such as *Escherichia coli* and *Helicobacter hepaticus*, induced by alcohol consumption while promoting the growth of butyrate-producing and commensal bacteria, including *Paramuribaculum intestinale* and *Bacteroides ovatus*. In conclusion, this study suggests that *A. muciniphila* restores ALD by regulating the gut microbiota, and this corrective effect is associated with alterations in the serum metabolism. Our research supplies a theoretical basis for developing *A. muciniphila* as an innovative generation of probiotic for preventing and managing ALD.

## 1. Introduction

Alcoholic liver disease (ALD) is a spectrum of liver injury that occurs due to chronic alcohol consumption [1,2]. According to the World Health Organization’s Global Status Report on Alcohol and Health, there has been a notable rise in alcohol consumption across various regions except the WHO European Region since 2000. This upward trend in alcohol consumption has led to a significant increase in both the prevalence and risk of alcoholic liver disease. These findings highlight the need for heightened attention and interventions aimed at addressing the growing burden of alcohol-related liver diseases [3]. However, the prevention and treatment options for ALD remain limited [4]. Furthermore, the U.S. Food and Drug Administration (FDA) has yet to grant direct approval for specific drugs and treatments designed explicitly for the treatment of patients with ALD [5]. Therefore, there is an urgent need to develop preventive and therapeutic approaches to ALD.

The etiology of ALD is multifactorial, involving intricate mechanisms that encompass various factors including but not limited to hepatic toxicity induced by alcohol and its metabolites, cell membrane lipid peroxidation, immune dysregulation, endotoxemia, and genetic predisposition [6]. In the meantime, an increasing number of studies have indicated a strong association between host metabolism, the homeostasis of gut microbiota, and ALD [7,8]. Emerging evidence strongly suggests that gut microbes are a critical target for preventing and treating ALD [9].

*Akkermansia muciniphila* is a Gram-negative anaerobic bacterium and colonizes in the human gastrointestinal (GI) tract with high abundance [10]. It induces mucin production which is necessary for intestinal integrity. *A. muciniphila* was first discovered and named by Dutch scholar Willem M. de Vos in 2004 in human feces [11]. *A. muciniphila* has been considered a promising next-generation probiotic due to its various beneficial physiological functions, such as immune regulation, maintenance of intestinal barrier function, modulation of mucus layer thickness, and anti-inflammatory effects [12,13,14]. Surprisingly, in less than 15 years, the beneficial effects of *A. muciniphila* have been translated from preclinical observations to human intervention in the context of metabolic syndrome. This situation is unique and unparalleled by other next-generation microorganisms [15,16]. Hence, *A. muciniphila* has wide application prospects.

Currently, research on *A. muciniphila* in relation to liver diseases has primarily focused on improving nonalcoholic liver disease [17,18,19,20]. There are only a few ALD-related studies compared to NAFLD (nonalcoholic fatty liver disease)-related studies. *A. muciniphila* has been shown to ameliorate ALD, and its mechanism of action includes strengthening the intestinal vascular barrier, restoring the abundance of *A. muciniphila* in the gut, increasing the thickness of the intestinal mucus layer, and the expression of tight junction proteins [21,22]. However, none of the aforementioned studies have comprehensively evaluated the alterations in host serum metabolism and gut microbial composition following interventions with *A. muciniphila* in the context of ALD. The protective mechanism of *A. muciniphila* in ALD still needs to be further explored. The aim of our study is to verify the improvement effect of *A. muciniphila* on alcoholic liver disease, and to examine the alterations of host serum metabolism and intestinal microorganisms after *A. muciniphila* intervention. This study may enhance our comprehension of the possible mechanisms that underlie the efficacy of *A. muciniphila* in treating ALD, thus supplying a theoretical basis for developing *A. muciniphila* as an innovative generation of probiotic for preventing and managing ALD.

## 2. Materials and Methods

### 2.1. Cultivation of A. muciniphila

*A. muciniphila* ATCC BAA-835 was cultured in brain heart infusion broth under strict anaerobic conditions for 48 h. A representative culture stock was used to determine the CFU/mL under anaerobic conditions by plate counting using mucin media containing 1% agarose. After centrifugation at 12,000× *g* for 10 min at 4 °C, the medium was removed, and *A. muciniphila* cells were washed with phosphate-buffered saline (PBS). The *A. muciniphila* cells were resuspended in PBS to a concentration of 2 × 10^8^ CFU/200 µL [23].

### 2.2. Animal Experiments

Specific pathogen-free C57BL/6 mice (female, age 10–12-week-old) were obtained from Shanghai Laboratory Animal Center (Shanghai, China). All experimental procedures commenced following the approval of the Institutional Animal Care and Use Committee of Shanghai Chedun Experimental Animal Breeding Farm (Approval number: ADO23003). All mice were housed in a controlled environment (20–22 °C, 40–60% humidity, 12 h light/dark cycle). Alcohol liver injury was induced in the mice using the NIAAA model with minor modifications [24]. After a 1-week acclimatization, the mice were fed a Lieber-DeCarli diet containing 1–5% vol% (TROPHIC Animal Feed High-tech Co., Ltd., Nantong, China) for 15 days [21,24]. The ethanol-fed or pair-fed mice were treated with *A. muciniphila* (2 × 10^8^ CFU/200 µL PBS) or PBS by intragastric infusion with a 24-gauge stainless steel free tube every other day, starting on day 1. On the last day of the experiment, the pair-fed mice received 5 g/kg maltose, and the ethanol-fed mice received 5 g/kg ethanol-water. Nine hours after the ethanol or maltose gavage, the mice were sacrificed. The blood was collected from the inferior vena cava and then centrifuged at 3000× *g* for 15 min at 4 °C for serum collection. Cecal contents and tissue samples of the cecum and colon were collected. Livers were carefully weighed and snap-frozen with liquid nitrogen. All the samples were stored at −80 °C immediately after collection for further analysis.

### 2.3. Biochemical Analyses

The biochemical markers, including serum alanine transaminase (ALT), aspartate aminotransferase (AST), and total bile acids (TBA), were measured by a fully automatic biochemical analyzer (Chemray 800, Rayto, Shenzhen, China). The liver tissues were pre-treated according to the instruction. The amounts of liver triglycerides were measured using a triglyceride quantification kit supplied by Nanjing Jiancheng Bioengineering Institute (Nanjing, China).

### 2.4. Histology and Immunohistochemistry (IHC)

The tissues of the liver samples were fixed in 10% neutral-buffered formalin for at least 24 h. Then, the tissues were embedded in paraffin and stained with hematoxylin and eosin (H&E) to assess the histologic features of steatosis and inflammation. Frozen liver tissues embedded in an optimal cutting temperature compound (Sakura, Torrance, CA, USA) were cut and stained with Oil Red O. Based on the observation using a laser-scanning confocal microscopy, the images were captured using a Nikon DSU3 camera (Nikon Corporation, Chiyoda Ward, Tokyo, Japan). For the immunofluorescence investigation of intestinal tight junction proteins, frozen intestinal sections were treated with anti-occludin and anti-claudin-2 antibodies (Thermo Fisher Scientific, Waltham, MA, USA), followed by labeling with a FITC-conjugated secondary antibody (Servicebio, Wuhan, China).

### 2.5. Serum Metabolites Assessment

The metabolomics study for plasma samples was based on the methods of our previous publication [25]. The serum metabolites were measured using an ultra-performance liquid chromatography linked to a tandem mass spectrometry (UPLC-MS/MS) system (ACQUITY UPLC-Xevo TQ-S, Waters Corp., Milford, MA, USA). The separation of the metabolites was achieved on a VanGuard pre-column (ACQUITY UPLC BEH C18, 2.1 mm × 5 mm, 1.7 μm) in combination with an analytical column (ACQUITY UPLC BEH C18, 2.1 mm × 100 mm, 1.7 μm). The column temperature and the manager temperature were set at 40 °C and 10 °C, respectively. Mobile phase A was water with formic acid (0.1%), and mobile phase B was acetonitrile/isopropanol (70:30). During gradient elution, the composition of the mobile phases changed in the following manner: keep 5% B for 1 min; increase to 78% B for 10 min; increase to 95% B for 2.5 min; increase to 100% B for 0.5 min; keep at 100% B for 2 min; rapidly drop to 5% B for 0.1 min; re-equilibrate at 5% B for 1.9 min. The injection volume was 5.0 μL, and the flow rate was 0.40 mL/min. Capillary voltages were 1.5 Kv in ESI+ and 2.0 Kv in ESI. The source temperature and desolvation temperature were set at 150 °C and 550 °C, respectively. The desolvation gas flow rate was 1000 L nitrogen per hour. The TMBQ program (v1.0, Metabo-Profile, Shanghai, China) was used to handle the raw data files produced by UPLC-MS/MS in order to perform peak integration, calibration, and quantitation for each metabolite. Discriminating metabolites between 2 classes of samples were identified using a statistically significant threshold of Variable Importance in Projection (VIP) value (VIP > 1), and further validated by a Student’s *t*-test analysis (*p* < 0.05).

### 2.6. Gut Microbiota Analysis

The genomic DNA from the cecal content were extracted using a TIANamp Micro DNA Kit (Tiangen, China). The 2bRAD-M library preparation basically followed the original protocol developed by Wang et al. [26] with minor modifications, where the endonuclease enzyme was changed from BsaXI enzyme to BcgI enzyme. Then, 4 U of the BcgI (NEB) enzyme was used to digest DNA (1–200 ng) for 3 h at 37 °C. The adaptors were then joined to the DNA fragments. The ligation reaction was carried out by mixing 5 µL of digested DNA with 10 µL of a ligation master mix that contained 800 U of T4 DNA ligase (NEB), 0.2 µm of each adaptor, and 5 µL of digested DNA. Ligation was carried out at 4 °C for 12 h. After that, ligation products were amplified, and PCR products were subjected to 8% polyacrylamide gel. Bands of about 100 bp were removed from the polyacrylamide gel, and the DNA was then diffused from the gel for 12 h at 4 °C in nuclease-free water. Using platform-specific barcode-bearing primers, PCR was used to add sample-specific barcodes. Each 20 µL PCR contained 25 ng of a gel-extracted PCR product, 0.2 µm of each primer, 0.3 mm dNTP, 1× Phusion HF buffer, and 0.4 U Phusion high-fidelity DNA polymerase (NEB). QIAquick PCR purification kit (Qiagen, Hilden, Germany) was used to clean up the PCR products before sequencing them on the Illumina Nova PE150 platform (Illumina, San Diego, CA, USA). Then, 2bRAD-M was executed at the Qingdao OE Biotech Co., Ltd. (Qingdao, China).

### 2.7. Statistical Analysis

The metabolome data were analyzed using the partial least square-discriminate analysis (PLS-DA), principal components analysis (PCA), and orthogonal partial Least squares-discriminant analysis (OPLS-DA) in SIMCAP+ (v.13.0; Umetrics, Umeå, Sweden). The self-developed platform iMAP (v1.0, Metabo-Profile, Shanghai, China) was used for the Circos plot and heatmap making. Principal co-ordinates analysis (PCoA; Bray–Curtis distance) was performed using R programing language. Oebiotech (Shanghai, China, https://www.oebiotech.com/, accessed on 6 May 2023) carried out alpha diversity analysis, beta diversity analysis, the random forest analysis, Phylogenetic Investigation of Communities by Reconstruction of Unobserved States (PICRUSt) analysis, and linear discriminant analysis Effect Size (LEfSe). The Tukey–Kramer method was used to assess the metabolome profile differences between the four groups in SPSS software (v.19.0, IBM Corp., Armonk, NY, USA). Mean ± SEM was used to plot the data.

## 3. Results

### 3.1. A. miciniphila Intervention Alleviates Alcohol-Induced Liver Injury

**Figure 1:**
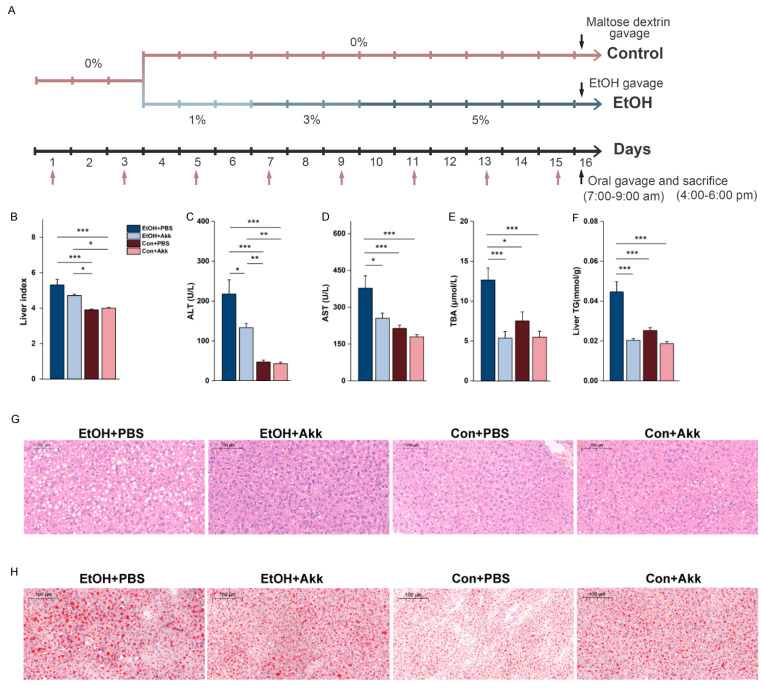
Effect of *A. muciniphila* on experimental ALD. (**A**) Experimental design diagram; pale pink arrows indicate *A. muciniphila* administration; (**B**) Liver index, i.e., liver-to-body-weight ratio; Levels of serum ALT (**C**), AST (**D**), and TBA (**E**); (**F**) Level of liver TG; (**G**) Representative photomicrographs of H&E liver sections; (**H**) Representative photomicrographs of Oil Red O-stained liver sections. Data are shown as mean ± SEM (n = 5–8 mice/group). * *p* < 0.05, ** *p* < 0.01, and *** *p* < 0.001.

To investigate the potential alleviating effect of *A. muciniphila* on alcohol-induced liver injury, we employed the NIAAA model with slight modifications and administered *A. muciniphila* through gavage every two days (Figure 1A). The intervention of *A. miciniphila* resulted in a decrease in the levels of the liver index, serum ALT, serum AST, serum TBA, and liver TG, which were all elevated due to ethanol treatment. Serum AST, serum TBA, and liver TG levels were even lower than those in the control group (Figure 1B–F). To further identify the liver injury, we conducted H&E staining of the liver tissue as well as Oil red O staining of the pathology sections for observation. By observing the H&E-stained sections, we found that EtOH+PBS mice exhibited infiltration of inflammatory cells in the liver tissue. In comparison to the Con+PBS group, the tissue showed enlarged vacuoles, indicating severe hepatic steatosis. However, the conditions were significantly improved by *A. muciniphila* (Figure 1G). Following Oil Red O staining, lipid droplets presented as red-stained areas. Ethanol treatment-induced enlargement of lipid droplets was suppressed by *A. muciniphila*, thereby inhibiting lipid deposition (Figure 1H). Collectively, these findings suggest that supplementation with *A. muciniphila* ameliorates alcohol-induced liver injury.

### 3.2. A. muciniphila Supplementation Alters Host Serum Metabolic Patterns

**Figure 2:**
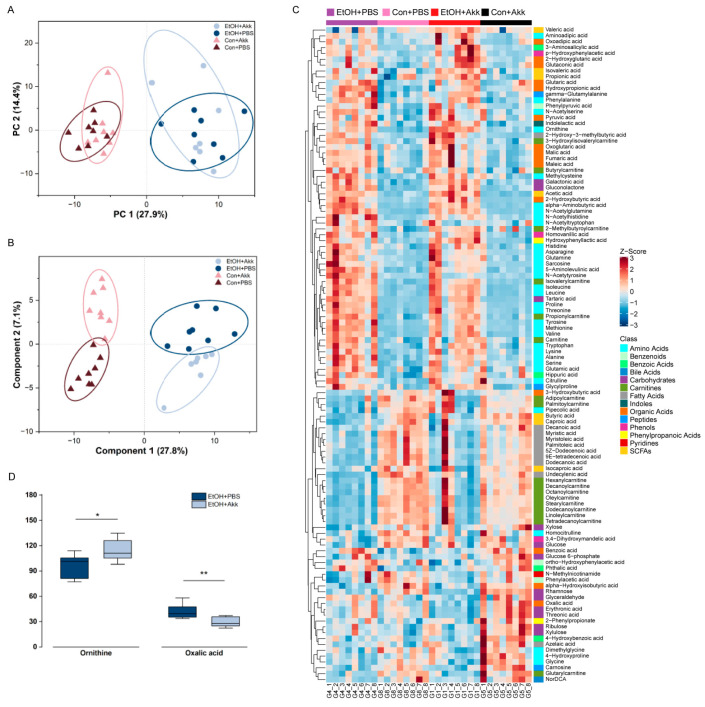
Effects of *A. muciniphila* interventions on the serum metabolism. (**A**) The PCA score plot and (**B**) PLS-DA score plot of metabolites from EtOH+Akk, EtOH+PBS, Con+Akk, and Con+PBS groups; (**C**) Clustering heatmap of the metabolism of the four groups; (**D**) Differential metabolite ornithine and oxalic acid content in groups EtOH+PBS and EtOH+Akk. * *p* < 0.05, ** *p* < 0.01.

**Figure 3:**
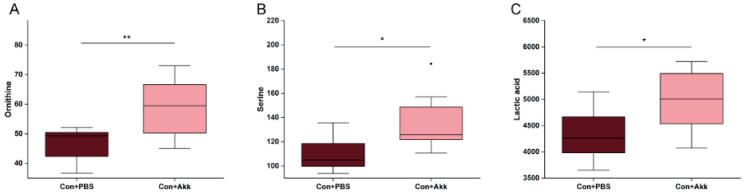
Comparison of differential metabolite content in E+A and E+P groups. (**A**) Content of ornithine; (**B**) Content of serine; (**C**) Content of lactic acid. * *p* < 0.05, ** *p* < 0.01.

To study how *A. muciniphila* intervention affects host metabolism, we analyzed the compositions of plasma metabolites using targeted metabolomics. First, principal component analysis (PCA) and partial least squares discriminant analysis (PLS-DA) were conducted to reveal a clear separation between the ethanol-treated group and the control group (Figure 2A,B). PCA showed a trend of altered metabolic patterns in the absence or presence of *A. muciniphila* (Figure 2A). PLS-DA revealed distinct serum metabolic patterns resulting from *A. muciniphila* and PBS-treated mice (Figure 2B). The metabolomics data analysis, using a clustering-based heatmap approach, revealed significant differences among groups after ethanol treatment or supplementation with *A. muciniphila* (Figure 2C). Metabolites responsible for the differences were identified using variable significance projection (VIP > 1) and Student’s *t* test (*p* < 0.05). We observed a significant increase in ornithine content in the serum of the ethanol-fed mice after *A. muciniphila* administration, accompanied by a marked decrease in oxalic acid content (Figure 2D). Additionally, the levels of ornithine, serine, and lactic acid increased noticeably in the Con+Akk group compared to the Con+PBS group (Figure 3). These results indicated that supplementation with *A. muciniphila* induced changes in the serum metabolic patterns.

### 3.3. A. muciniphila Administration Restores Intestinal Homeostasis

**Figure 4:**
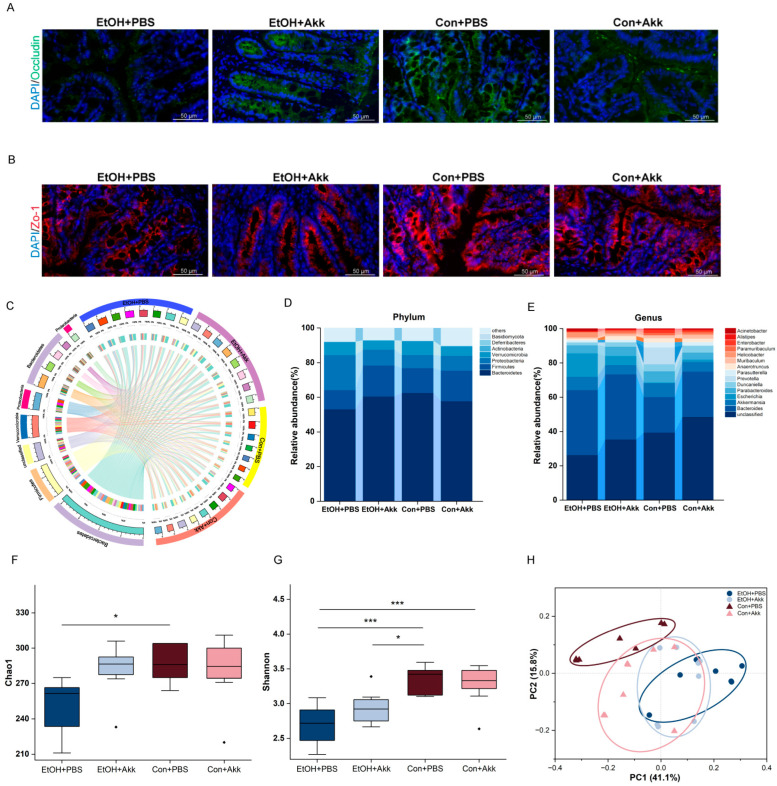
Effects of *A. muciniphila* intervention on intestinal barrier and gut microbiota in mice. (**A**) Immunofluorescence staining of occludin in the colon; (**B**) Immunofluorescence staining of Zo-1 in the colon; (**C**) The Circos plot showing the composition of gut microbiota in each group; (**D**) Mean relative abundance of the most abundant phyla and (**E**) genera in the cohort population gut microbiome; (**F**) Alpha diversity analysis using Chao1 index and (**G**) Shannon index; (**H**) Beta diversity analysis using PCoA of the gut microbiota based on Bray–Curtis dissimilarities. * *p* < 0.05 and *** *p* < 0.001.

The intestinal barrier is fundamental for modulating intestinal homeostasis and is the body’s first line of defense against pathogen invasion. Immunofluorescence staining was introduced to detect the expressions of occludin and Zo-1. In contrast to the mice fed ethanol as controls, we found recovered immunoreactivity of occludin and tight junction protein 1 (Zo-1) in colonic epithelial cells from *A. muciniphila*-treated mice (Figure 4A,B).

Gut microbiota plays an important role in the development of ALD. To examine the alterations in gut microbial composition in the mice with ALD following the ingestion of *A. miciniphila*, we analyzed the composition of gut microbiota in the cecal contents of the mice using 2bRAD-M. A total of 702 microorganism species were identified in all samples. At the phylum level, Bacteroidetes, Firmicutes, Proteobacteria, and Verrucomicrobia were the most predominant taxa (Figure 4C). The proportion of Proteobacteria was increased and the proportions of Bacteroides and Firmicutes were decreased compared to the Con+PBS group. But the changes were reversed by the treatment of *A. muciniphila* (Figure 4D). At the genus level, unclassified *Bacteroides*, *Akkermansia*, *Escherichia*, and *Parabacteroides* were the most abundant genera (Figure 4E). In addition, we assessed the effect of *A. muciniphila* administration on the diversity of gut microbiota. The alpha diversity was assessed by Chao1 index and Shannon index. Ethanol treatment significantly decreased the alpha diversity index (Chao1 and Shannon). Descriptively, there was an increase in alpha diversity with *A. muciniphila*, although this increase did not reach statistical significance (Figure 4F,G). Beta diversity was conducted using PCoA. As is shown in Figure 4F, the gut microbiota communities of the mice fed with ethanol diet were remarkably different from the control diet group. Meanwhile, PCoA revealed a clear pattern of separation between the gut microbiota communities in the mice treated with *A. muciniphila* or PBS (Figure 4H). Thus far, we have successfully demonstrated the ability of *A. muciniphila* to alter the composition of the gut microbiota.

### 3.4. Biomarkers of the Gut Microbiome in Different Groups

**Figure 5:**
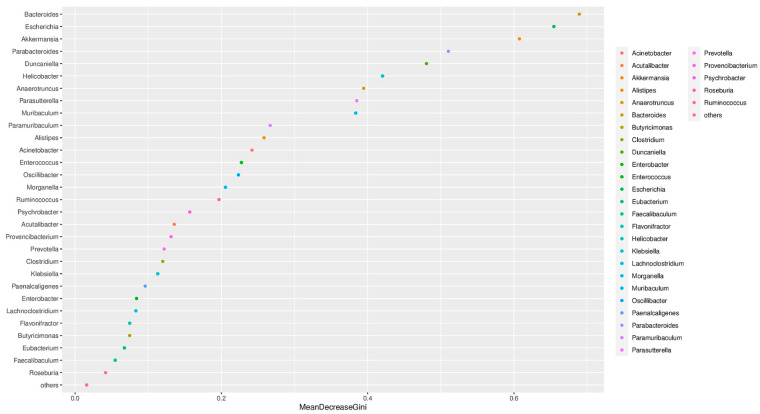
The top 30 most important microbial genera determination of microbiota structure with *A. muciniphila* administration were obtained by using random forest analysis.

**Figure 6:**
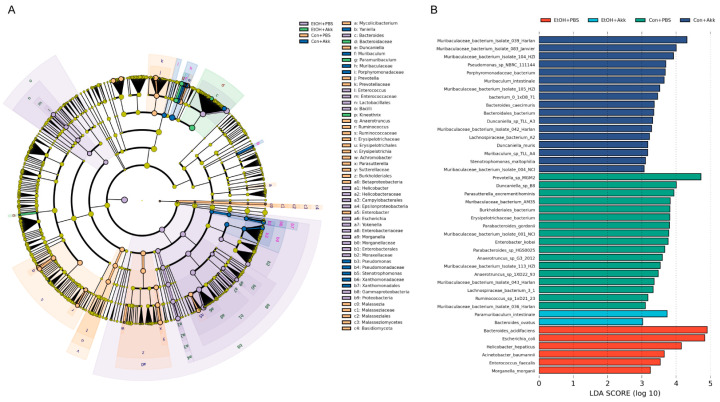
LEfSe analysis of the key biomarkers in different groups. (**A**) LEfSe analysis from phylum to genus; (**B**) LDA histogram of significant microbial species (LDA score > 3).

To identify the specific microorganisms responsible for these differences, we performed random forest and LEfSe analyses. The random forest algorithm was used to rank the microbial traits that contribute to the gut microbiota restructuring process. Using the random forest modeling, we obtained the top 30 most important microbial genera for determining microbiota structure with *A. muciniphila* administration (Figure 5). LEfSe was used to identify microbial taxa with significant differences in abundance between groups [27]. Ethanol feeding not only enriched *Bacteroides* and *Enterococcus* but also led to a marked enrichment of the pathogenic bacterial taxa *Helicobacter*, *Escherichia*, *Yokenella,* and *Morganella* in mice. Additionally, *Paramuribaculum* and *Kineothrix* were unique genera found only in the EtOH+Akk group (Figure 6A). At the species level, the abundance of *Paramuribaculum intestinale* and *Bacteroides ovatus* was significantly increased in the EtOH+Akk group, whereas the abundance of *Bacteroides acidifaciens*, *Escherichia coli*, *Helicobacter hepaticus*, *Acinetobacter baumannii*, *Enterococcus faecalis,* and *Morganella morganii* was significantly decreased (Figure 6B). Taken together, these results indicate that *A. muciniphila* has the ability to reduce the abundance of alcohol-induced harmful microbiota and increase the presence of intestinal commensal bacteria in mice with ALD.

### 3.5. A. muciniphila Administration Alters Intestinal Homeostasis

**Figure 7:**
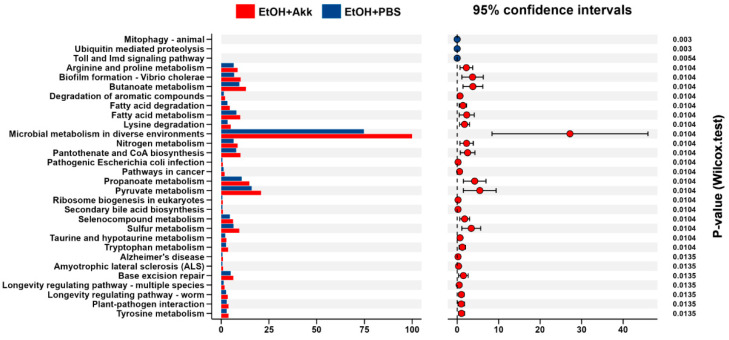
Differences in the general functional characteristics of the gut microbiota. Bacterial functional pathways were estimated by using the PICRUSt algorithm.

To further explore the biological functions, the most different top thirty KEGG pathways between EtOH+Akk and EtOH+PBS comparisons were generated by the PICRUSt algorithm (Figure 7). *A. muciniphila* administration upregulated many pathways, including arginine and signaling pathways, biofilm formation, butanoate metabolism, degradation of aromatic compounds, fatty acid degradation, etc. This result indicated that *A. muciniphila* altered the functional capabilities of the intestinal microbial community in mice fed with ethanol.

## 4. Discussion

**Figure 8:**
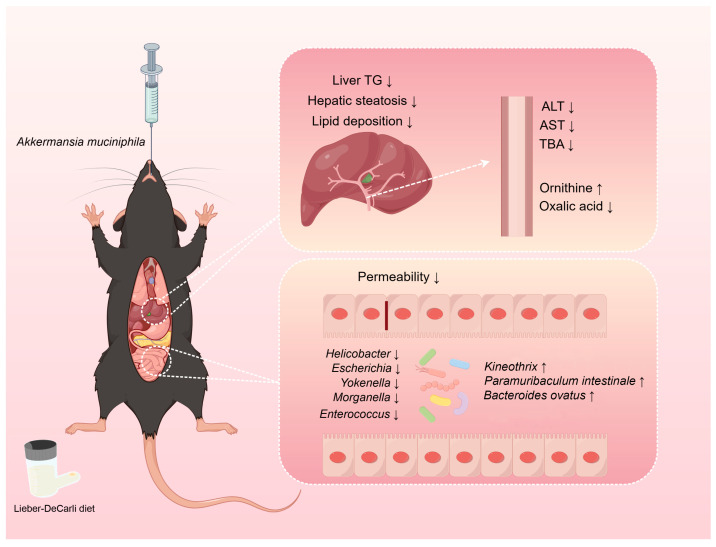
Diagram of the mechanism by which *A. muciniphila* improves ALD. *A. muciniphila* improves ALD by altering host metabolism and regulating the gut microbiota. “↑” represents increase and “↓” represents decrease.

Recent studies on ALD have found that several natural extracts or bioactive compounds exert their effects by augmenting the abundance of *A. muciniphila* [28,29,30,31,32]. This finding highlights the potential of *A. muciniphila* as a viable treatment option for alcoholic liver disease. A number of studies have demonstrated the association between *A. muciniphila* and ALD [21,22,33]. However, the influence of *A. muciniphila* supplementation in modulating serum metabolites and gut microbiota in mice with ALD remains unclear. In the present study, we validated the ameliorative effect of *A. muciniphila* on ALD. Furthermore, we explored potential mechanisms underlying the effects of *A. muciniphila* on ALD beyond its role in improving intestinal barrier function. Our study integrated the assessment of host serum metabolic patterns and gut microbiota, which have been overlooked in similar studies. Our study revealed that *A. muciniphila* ameliorates ALD during prolonged consumption by modulating host serum metabolism and remodeling the gut microbial community (Figure 8).

Phenotyping, biochemical parameters, and histopathological examination methods were used to confirm the effect of *A. muciniphila*. The consumption of *A. muciniphila* reduced the levels of liver index, serum ALT, AST, TBA, and liver TG. It also showed inhibitory effects on pathological changes in liver tissue, reducing the extent of fatty degeneration and lipid deposition. Alcohol consumption disrupts metabolic pathways in cells, leading to liver cell damage. ALT and AST are normally stored in hepatocytes, and damage to hepatocytes can result in elevated serum ALT and AST levels [34]. At the same time, the hepatocyte metabolism is disturbed, and insulin signaling transduction is impaired, resulting in increased TG storage in liver cells [35]. Changes in mouse serum TBA may also imply changes in host metabolism. Hepatic steatosis is one of the hallmarks of alcoholic liver disease. ALD often progresses from simple steatosis through various stages of steatohepatitis, fibrosis, cirrhosis, and ultimately liver failure, all of which manifest liver dysfunction [36]. Our study found that *A. muciniphila* intervention during persistent alcohol consumption effectively suppressed the development of alcoholic fatty liver.

To elucidate the underlying mechanisms responsible for the protective effects of *A. muciniphila*, we used targeted metabolomics to analyze the variation of serum metabolites in mice. We found that *A. muciniphila* could alter host serum metabolic patterns. PCA showed a trend of altered metabolic patterns in the absence or presence of *A. muciniphila,* and PLS-DA revealed distinct serum metabolic patterns resulting from *A. muciniphila* and PBS-treated mice. Two important differential metabolites, ornithine and oxalic acid, were screened using multidimensional statistics combined with unidimensional statistical analysis. Supplementation with *A. muciniphila* increased ornithine levels and decreased oxalic acid levels in mice with ALD. Ornithine is thought to be beneficial for gut heath [37]. Gut microbiota regulates the homeostasis of the intestinal mucosal layer via intestinal immune cells, which can influence the mucosal cell proliferation and mucin production [38]. L-Ornithine increases the levels of the aryl hydrocarbon receptor ligand L-kynurenine produced from tryptophan metabolism in gut epithelial cells, which in turn increases gut immune cells [39]. Ornithine aspartate is thought to have some hepatoprotective properties in patients with fatty liver disease [40]. Prolonged elevation of serum oxalic acid levels has been associated with the development of liver cirrhosis [41]. Therefore, elevated serum levels of oxalic acid are considered as a detrimental indicator of liver health deterioration. We believe that *A. muciniphila* probably exerts its protective effect against alcohol-induced damage by increasing the production of metabolites that promote intestinal homeostasis while reducing the levels of harmful substances present in the host serum.

Ethanol disrupts the intestinal barrier and dysregulates intestinal ecology [42]. Ethanol and its metabolite, acetaldehyde, can directly or indirectly damage intestinal epithelial cells, thereby consequently compromising the integrity of the intestinal mucosal barrier. Furthermore, chronic alcohol consumption induces a downregulation in the expression of proteins involved in the regulation of tight junction proteins, resulting in the decreased expression of tight junction proteins between intestinal epithelial cells [43]. Both animal models and clinical evidence suggest that alcohol consumption disrupts the composition and structure of the gut microbiota, leading to a decrease in the abundance of Firmicutes (Gram-positive bacteria) and Lactobacillus (lactic acid-producing bacteria) in the gut [44]. Ethanol consumption disrupts the intestinal epithelial barrier, resulting in the transfer of bacteria or bacterial components to the liver, leading to liver damage [45]. Although the pathogenesis of ALD is complex, the maintenance of intestinal barrier function and the composition of the gut microbiota are still recognized to play crucial roles in the pathogenesis of ALD [9].

Given the close physiological relationship between the intestine and liver, intestinal barrier function is critical for liver homeostasis, and disruption of intestinal barrier integrity can accelerate the development of liver disease [46]. To investigate how *A. muciniphila* affects intestinal permeability, we used immunofluorescence staining to detect the expression of occludin and Zo-1. Zo-1 is an important component of the intestinal barrier [47]. Occludin is a transmembrane protein that is depleted by ethanol in the colon. Occludin deficiency has been suggested to promote the ethanol-induced disruption of colonic epithelial junctions, intestinal barrier dysfunction, and liver damage in mice [48]. Sesamol and apigenin could increase the expression of Occludin, claudin-1, and Zo-1 to attenuate colitis [49,50]. *A. muciniphila* has been shown to attenuate ethanol-induced damage by restoring intestinal barrier function [21]. This finding was confirmed in our study. *A. muciniphila* alleviated the alcohol-induced reduction of the colonic tight junction proteins Occludin and ZO-1, and it reduced the degree of intestinal barrier damage. Our study confirmed this result [21]. 

Unsurprisingly, in our study, ethanol consumption led to gut microbiota dysbiosis in mice, but the supplementation with *A. muciniphila* inhibited the dysbiosis. Ethanol consumption significantly decreased the alpha diversity. Alpha diversity increased as *A. muciniphila* treatment increased, although it did not reach statistical significance. PCoA showed that the intestinal microbial community was altered following *A. muciniphila* administration.

We then used random forest and LEfSe analysis to identify the microbes responsible for these discrepancies. At the species level, the abundance of *Paramuribaculum intestinale* and *Bacteroides ovatus* was significantly increased in the EtOH+Akk group, while that of *Bacteroides acidifaciens*, *Escherichia coli*, *Helicobacter hepaticus*, *Acinetobacter baumannii*, *Enterococcus faecalis*, and *Morganella morganii* was significantly decreased. Interestingly, *Escherichia coli*, *Helicobacter hepaticus*, and *Morganella morganii* are considered to be pathogenic and belong to phyla Proteobacteria [51,52,53,54]. The expansion of Proteobacteria is a microbial signature of dysbiosis in the gut microbiota [55]. Both animal and clinical trials have shown a remarkable increase in the abundance of Proteobacteria during the process of ALD [56]. However, *A. muciniphila* supplementation decreased the relative abundance of Proteobacteria while promoting the relative abundance of Firmicutes and Bacteroidetes. *Kineothrix* belongs to Firmicutes phylum, a butyrate-producing bacterium [57]. An increase in the relative abundance of *Kineothrix alysoides* correlates with a decrease in anxiety-like behaviors [58]. Butyrate has anti-inflammatory effects and can improve intestinal barrier function and mucosal immunity [59]. Butyrate is a critical factor in maintaining gut health [60]. Butyrate is able to support colonocyte function, reduce inflammation, maintain the gut barrier, and promote a beneficial microbiome [61]. Moreover, butyrate has been reported to have the ability to inhibit liver injury in a mouse model of NAFLD [62]. However, its role in ALD remains unclear. *A. muciniphila* may be effective in the progression of ALD by increasing the abundance of the butyrate-producing microorganisms in the intestine, thereby increasing intestinal levels of butyrate. Unfortunately, our study lacks further experimental validation. There are few studies on *Paramuribaculum intestinale*. Existing studies have found a positive correlation between *Paramuribaculum* abundance and the severity of Epstein–Barr virus (EBV) infection and colitis, but there is a higher abundance in kidney injury [63,64,65]. There was also a significant enrichment of bacteria in the EtOH+Akk group, *Bacteroides ovatus*, which is characterized as one of the predominant species in intestinal commensal bacteria [66]. The results suggest that *A. muciniphila* may be a modulator in the development of ALD by inhibiting the ethanol-induced proliferation of pathogenic bacteria and promoting the abundance of commensal bacteria in the gut microbiota.

## 5. Conclusions

Overall, our study demonstrated the ability of *A. muciniphila* to ameliorate ethanol-induced injury in mice models of ALD, which differ from those employed in previous studies, and it established, for the first time, a correlation between the beneficial effect of *A. muciniphila* on ALD and alterations in host metabolism and gut microbiota composition. We found that the beneficial effect of *A. muciniphila* on ALD was associated with changes in host metabolism. Our study also revealed that *A. muciniphila* inhibited the proliferation of harmful bacteria and promoted beneficial effects, effectively regulating the gut microbiota and thereby alleviating ALD. This study adds to the mechanisms underlying the ameliorative effects of *A. muciniphila* on ALD and provides a theoretical basis for the future application of *A. muciniphila* as a therapeutic modality for ALD.

## Data Availability

All the data in this study are contained within the manuscript.
